# Characterization of Protection Afforded by a Bivalent Virus-Like Particle Vaccine against Bluetongue Virus Serotypes 1 and 4 in Sheep

**DOI:** 10.1371/journal.pone.0026666

**Published:** 2011-10-21

**Authors:** Ana Cristina Pérez de Diego, Thimmasandra N. Athmaram, Meredith Stewart, Belén Rodríguez-Sánchez, José Manuel Sánchez-Vizcaíno, Robert Noad, Polly Roy

**Affiliations:** 1 Department of Infectious Diseases, London School of Hygiene and Tropical Medicine, London, United Kingdom; 2 Centre for Animal Health Surveillance, Complutense University of Madrid, Madrid, Spain; University of Kansas Medical Center, United States of America

## Abstract

**Background:**

Bluetongue virus (BTV) is an economically important, arthropod borne, emerging pathogen in Europe, causing disease mainly in sheep and cattle. Routine vaccination for bluetongue would require the ability to distinguish between vaccinated and infected individuals (DIVA). Current vaccines are effective but are not DIVA. Virus-like particles (VLPs) are highly immunogenic structural mimics of virus particles, that only contain a subset of the proteins present in a natural infection. VLPs therefore offer the potential for the development of DIVA compatible bluetongue vaccines.

**Methodology/Principal Findings:**

Merino sheep were vaccinated with either monovalent BTV-1 VLPs or a bivalent mixture of BTV-1 VLPs and BTV-4 VLPs, and challenged with virulent BTV-1 or BTV-4. Animals were monitored for clinical signs, antibody responses, and viral RNA. 19/20 animals vaccinated with BTV-1 VLPs either alone or in combination with BTV-4 VLPs developed neutralizing antibodies to BTV-1, and group specific antibodies to BTV VP7. The one animal that showed no detectable neutralizing antibodies, or group specific antibodies, had detectable viral RNA following challenge but did not display any clinical signs on challenge with virulent BTV-1. In contrast, all control animals' demonstrated classical clinical signs for bluetongue on challenge with the same virus. Six animals were vaccinated with bivalent vaccine and challenged with virulent BTV-4, two of these animals had detectable viral levels of viral RNA, and one of these showed clinical signs consistent with BTV infection and died.

**Conclusions:**

There is good evidence that BTV-1 VLPs delivered as monovalent or bivalent immunogen protect from bluetongue disease on challenge with virulent BTV-1. However, it is possible that there is some interference in protective response for BTV-4 in the bivalent BTV-1 and BTV-4 VLP vaccine. This raises the question of whether all combinations of bivalent BTV vaccines are possible, or if immunodominance of particular serotypes could interfere with vaccine efficacy.

## Introduction

Bluetongue is a vector-borne disease of ruminants caused by a double-stranded RNA (dsRNA) virus of the genus *Orbivirus* in the family *Reoviridae.* In southern Africa, where bluetongue is endemic, bluetongue virus (BTV) cycles between midges of the *Culicoides* genus and wild and domestic ruminants [1]. In livestock, sheep and cattle can both be affected but sheep generally show the most severe clinical signs [2], [3], [4]. Historically, 24 different serotypes of BTV have been characterized. In addition, Toggenburg virus was described in 2008 and is considered as a putative 25^th^ BTV serotype [5], [6], and there has been a recent report of a 26^th^ serotype in Kuwait [7]. Before 1998, outbreaks of bluetongue in Europe were sporadic and relatively small scale. However, since then there have been sustained and repeated incursions into the continent of different serotypes that have had substantial economic, political and animal welfare impacts [8], [9], [10], [11], [12], [13]. A consequence of these outbreaks has been a renewed interest in the development of vaccines to BTV.

Vaccination is an effective measure to control bluetongue disease [10]; immunisation with a number of different vaccines including attenuated virus, inactivated virus, pox-based vaccines and recombinant protein immunogens result in the induction of neutralising antibodies and protection against disease and viraemia [3], [10], [11], [14]. One of the vaccine approaches is the production of BTV virus like particles (VLPs). VLPs are non-infectious mimics of the virus formed from expression of only virus structural proteins in a heterologous expression system [14], [15], [16]. As these particles do not contain viral genetic material and their production does not involved the expression of viral transcription complex or non-structural protein they are inherently safe and are compatible with the need to distinguish between infected and vaccinated animals (DIVA). This is important because one of the barriers to routine vaccination for livestock disease is the need to trade between areas where the virus is endemic and areas where it is exotic [17]. In the case of BTV, construction of VLP involves the co-expression of four structural proteins, VP2, VP3, VP5 and VP7 to form a multi-layered particle. VP3 and VP7 form a core structure which is relatively invariant between serotypes, VP7 is used as a group-specific antigen for BTV [18]. VP2 and VP5 form the virus outer capsid, which is responsible for virus attachment and penetration of host cells, VP2 is the serotype determinant [1], [18].

BTV protection is serotype specific; immunization with one of the 26 BTV serotypes does not elicit a high cross-protection against other serotypes. Successful recombinant or inactivated bivalent and polyvalent vaccines have been described for BTV that include serotypes 2 and 4 [19], and serotypes 1, 2, 10, 13 and 17 [20]. The strategy behind such multivalent vaccines is that a cocktail of immunogens to different serotypes will elicit multiple serotype-specific responses or cross-protective responses. In this study we test the protective efficacy of a cocktail of BTV VLPs for BTV-1 and BTV-4 in challenge experiments in Merino sheep, with the aim of validating that this combination of VLPs provided protective responses to both viruses. Although there was complete protection from clinical disease with challenge for BTV-1, there was some evidence that there was less complete protection for BTV-4 with the bivalent vaccine.

## Results

### Development of group specific antibodies in vaccinated animals

**Figure 1:**
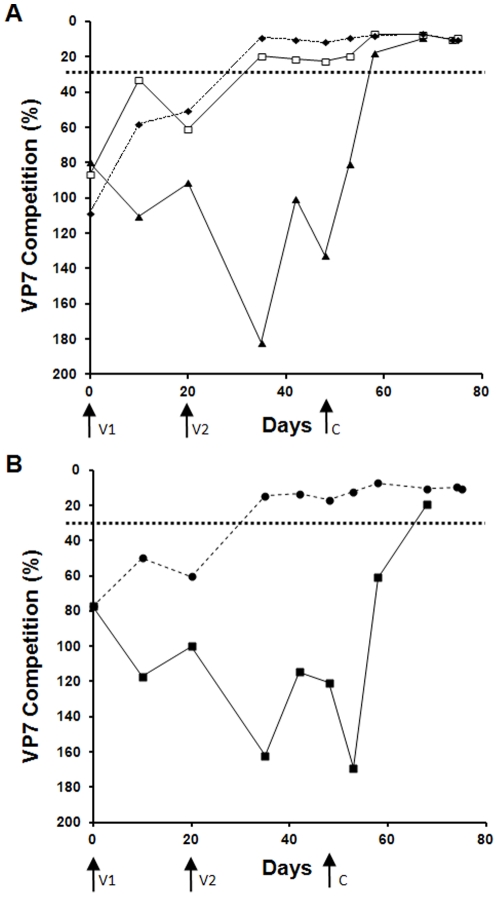
Group specific immune response of sheep vaccinated with VLPs. Sero-conversion of vaccinated sheep was monitored using competitive ELISA (POURQUIER® Bluetongue Competitive ELISA) specific to VP7, the BTV group specific core antigen. V1 and V2 indicate the day sheep were vaccinated and boosted with immunogen, respectively. C indicates the day sheep were challenged with virulent BTV. The data represented is the average of all animals in each group, no adjustments or omissions have been made. The threshold for the sample to be considered as seropositive for VP7 was 35%. **A**. ELISA results of groups of animals were immunised with VLPs and challenged with virulent BTV-1. All vaccinated animals, except A2 had a BTV group specific antigen response above threshold as determined by the kit prior to challenge. None of the control animals had a response prior to challenge. Average results of groups of sheep vaccinated with different immunogens are shown: Group D (control:▴), Group A (BTV-1 VLPs:⧫) and Group B (BTV-1 & BTV-4 VLPs:□). **B.** ELISA results of groups of animals vaccinated with VLPs and challenged with virulent BTV-4. All vaccinated animals had a BTV group specific antigen response prior to challenge. None of the control animals had a response prior to challenge. Average results of groups of sheep vaccinated with different immunogens are shown: Group E, (saline+adjuvant: ▪) and Group C (BTV-1 VLPs & BTV-4 VLPs: •).

A total of 26 sheep were divided into 5 groups (A–E) and immunised with monovalent BTV-1 VLPs (Group A), bivalent BTV-1 and BTV-4 VLPs (Groups B and C) or saline plus adjuvant only (Groups D and E). In all cases the sheep were immunised subcutaneously on day 0 and again on day 20 (as Materials and Methods). The development of a group specific anti-BTV (anti-VP7) response was monitored in all animals initially using a commercial competitive ELISA kit (IDVET; Fig. 1A and 1B). No animal showed a positive result for VP7 recognition at the start of the experiment (day 0). The results from the competitive ELISA test showed that only 7 of the 20 VLP vaccinated animals were positive on the day of the booster immunisation (day 21). After the second vaccination, 19 of the 20 sheep vaccinated sero-converted after 2 weeks of the booster-vaccination (day 35). None of control animals developed antibodies to BTV VP7 prior to challenge with virulent virus, remaining above the threshold of 35% VP7 Competition. One VLP vaccinated sheep (sheep A2), did not develop any positive competitive ELISA result for BTV group specific antigen until 10 days after challenge (day 58). This timing is consistent with saline vaccinated control sheep challenged with the same virus (i.e., BTV-1; Group D), where all 3 sheep first became positive for BTV group specific antigen using the same test on day 58 (Fig. 1A). All animals in Group C (BTV-1 and BTV-4 VLP) raised antibody against the group specific antigen and sero-converted prior to challenge with virulent BTV-4, while the Group E (control) were sero-converted only after challenge as expected (Fig. 1B). The results from competitive ELISA were validated using an alternate commercially available, a double recognition ELISA test (Ingenasa). In contrast to the competition ELISA results, all sera from animals vaccinated with VLPs (groups A–C), were positive for BTV group specific antigen from 10 days after the first immunisation and remained positive for the rest of the experiment.

### Development of neutralisation antibodies in to BTV-1 and BTV-4 in VLP vaccinated sheep

**Figure 2:**
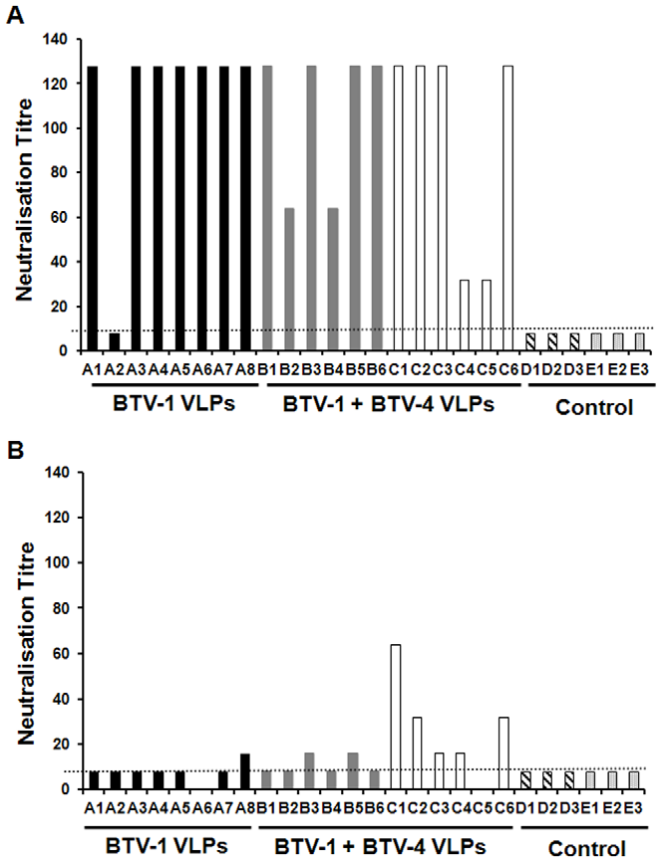
Neutralisation antibody response of sheep vaccinated with VLPs. Virus neutralisation antibody titres of individual sheep vaccinated with BTV-1 VLP (black), BTV-1 & BTV-4 VLP (grey), BTV-1 & BTV-4 VLP (white) and non-vaccinated sheep (black and white striped or spotted bars) prior to challenge with virulent virus as determined by the neutralisation of 50 TCID_50_. The group of each animal is indicated by an A, B, C, D or E alongside the animal number. The serum neutralisation titres against BTV-4 were not determined for animals A6 (Group A) and C5 (Group C) due to lack of serum collected. **A**. Neutralisation antibodies elicited against BTV-1. **B**. Neutralisation antibodies elicited against BTV-4. Titres below the threshold of the assay (dashed line) were considered to be negative.

Although ELISA to detect antibodies to VP7 is indicative of an immune response to the VLP immunogens, it is not possible to distinguish between the response to BTV-1 VLPs and BTV-4 VLPs with this test. VP7 is present in both VLPs and antibodies to this protein would be cross-reactive. To assess serotype specific immune responses to VLPs, we carried out virus neutralisation tests to determine if the serotype specific neutralising antibodies were raised in vaccinated sheep (Fig. 2). With the exception of sheep A2, all animals vaccinated with monovalent BTV-1 VLPs (Group A) or bivalent BTV-1 and BTV-4 VLPs (Groups B and C) had serum neutralising titres of at least 32, with a median titre of 128 (Fig. 2A). Neutralisation of BTV-1 by sheep A2 serum was indistinguishable from background. Incidentally, sheep A2 also did not develop an immune response to VP7 until after challenge. The neutralisation antibody titres to BTV-1 were consistently higher than those observed to BTV-4. This was highlighted by virus neutralisation titres of 16 or below for BTV-4 in 7 of the 12 sheep vaccinated with bivalent immunogens. The remaining 4 of this group had neutralisation titres ranged from 32 to 64, however, due to insufficient serum collected from animal C5, it was not possible to determine whether it had raised neutralisation antibody titre (Fig. 2B). The median neutralisation titre of BTV-4 for these animals was 16. Surprisingly, one of the sheep vaccinated with BTV-1 VLPs (sheep A8) also had neutralisation antibodies against BTV-4, although at a low titre of 16 (Fig. 2B).

Due to low antibody titres for BTV-4 neutralisation using the 50 TCID_50_ method, virus neutralisation for groups B and C was further confirmed by 50% plaque reduction assay which showed similar level of antibody titres.

### Clinical signs in sheep following challenge with virulent virus

**Figure 3:**
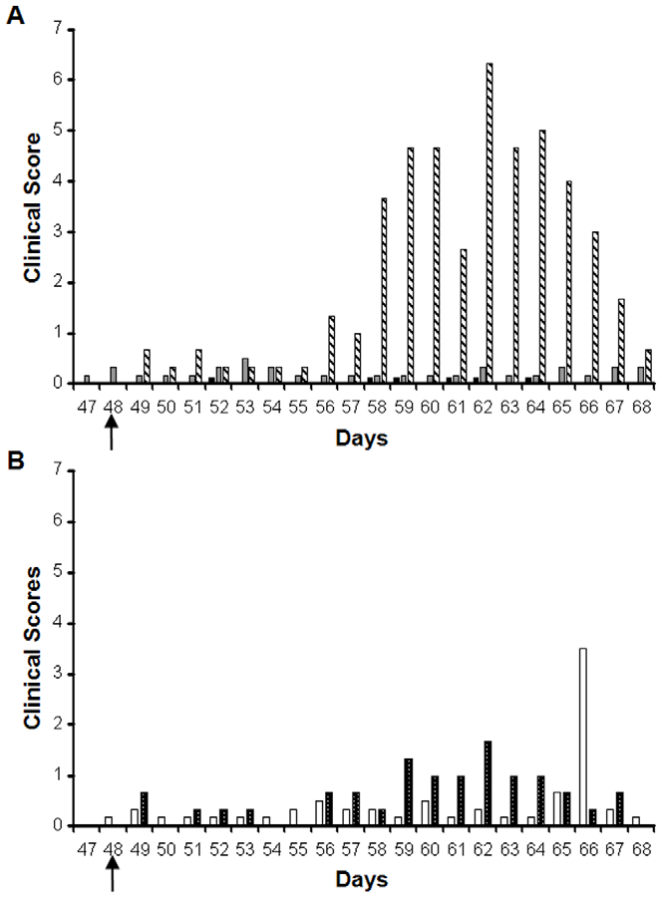
The average clinical score of sheep challenged with virulent BTV-1 (A) or BTV-4 (B). All sheep vaccinated with BTV-1 VLPs (Group A; black bar), BTV-1 & BTV-4 VLP (Group B; grey bar) and challenged with virulent BTV-1 were protected from detectable disease. All control sheep challenged with BTV-1 (Group D; striped bar) developed clinical signs of bluetongue disease. Sheep vaccinated with BTV-1 and BTV-4 VLPs (Group C; white bar) and challenged with BTV-4 show some clinical score on this chart because of one animal (C5). No other animal in the group showed clinical signs of bluetongue. The control animals challenged with BTV-4 (Group E; spotted bars) developed mild clinical bluetongue. Individual sheep were scored on a standard, 8 point clinical reaction scale. Data presented in the chart is the average score for each group.

**Figure 4:**
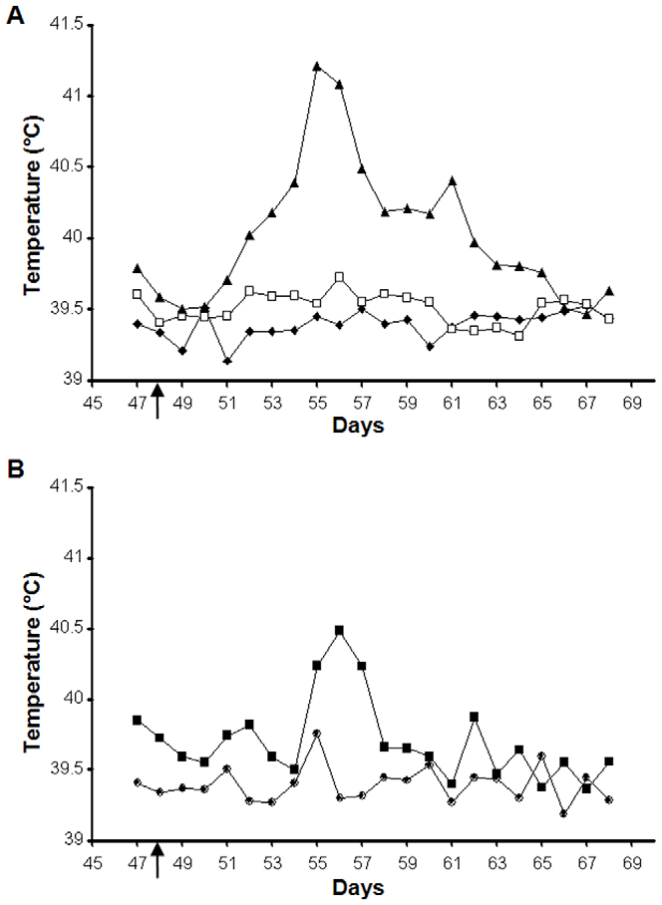
Temperature response of sheep challenged with virulent BTV. The average rectal temperatures (°C) of BTV-1 VLP (Group A) and BTV-1 & BTV -4 VLP (Group B and C) vaccinated and saline vaccinated sheep (Group D and E) were monitored after challenge. The time of challenge is indicated by an arrow. All animals in each group were included in the analysis. **A.** Animals challenged with virulent BTV-1. The different groups are indicated; Group D (▴), Group A (⧫) and Group B (□). **B.** Animals challenged with virulent BTV-4. The different groups are indicated; Group E (▪) and Group C (•).

As a majority of the sheep vaccinated with VLPs had demonstrated a group specific or neutralising antibody response, sheep in all groups were challenged on day 48 with virulent virus. Groups A, B and D were challenged with BTV-1, groups C and E were challenged with BTV-4. All animals in control groups D and E (6/6 animals), showed clinical signs that were consistent with infection with BTV from day 58 (Fig. 3A and 3B), with the most severe visible clinical signs observed in animals challenged with virulent BTV-1 (Fig. 3A). In contrast, none of the animals vaccinated with monovalent or bivalent immunogens containing BTV-1 VLPs (groups A and B) showed any detectable gross clinical signs or increased temperature (Fig. 4). Only the control animals had an increase in rectal temperature after challenge with virulent BTV-1. The febrile response in the control animals lasted greater than 8 days, with temperatures failing to return to normal during the course of the experiment (Fig. 4A). No increase in rectal temperature was recorded in any of the animals that were immunised with BTV-1 VLPs or BTV-1 and BTV-4 VLPs after challenge with BTV-1 (Fig. 4A). Control animals in group D challenged with BTV-1 showed pyrexia from day 54 to day 61, but animals in VLP groups A and B, including sheep A2, had no detectable pyrexia in this period. With the parallel, challenge of groups C and E with BTV-4, results were quite different. The control group animals (group E) displayed clinical signs for a short duration of time and an increase in rectal temperature above normal between days 55 and 57 (Fig. 4B). For the animals vaccinated with the bivalent vaccine (group C) 5/6 animals showed no clinical signs (Fig. 3B) including no increase in body temperature over the challenge study (Fig. 4B). However, one animal, C5 had a transient increase in rectal temperature between days 54 and 56 and on day 65. This animal also showed apathy, mandibular oedema, hypersalivation and dyspnoea on day 65 and died on day 66.

### Detection of viral RNA of challenge virus in vaccinated animals

**Figure 5:**
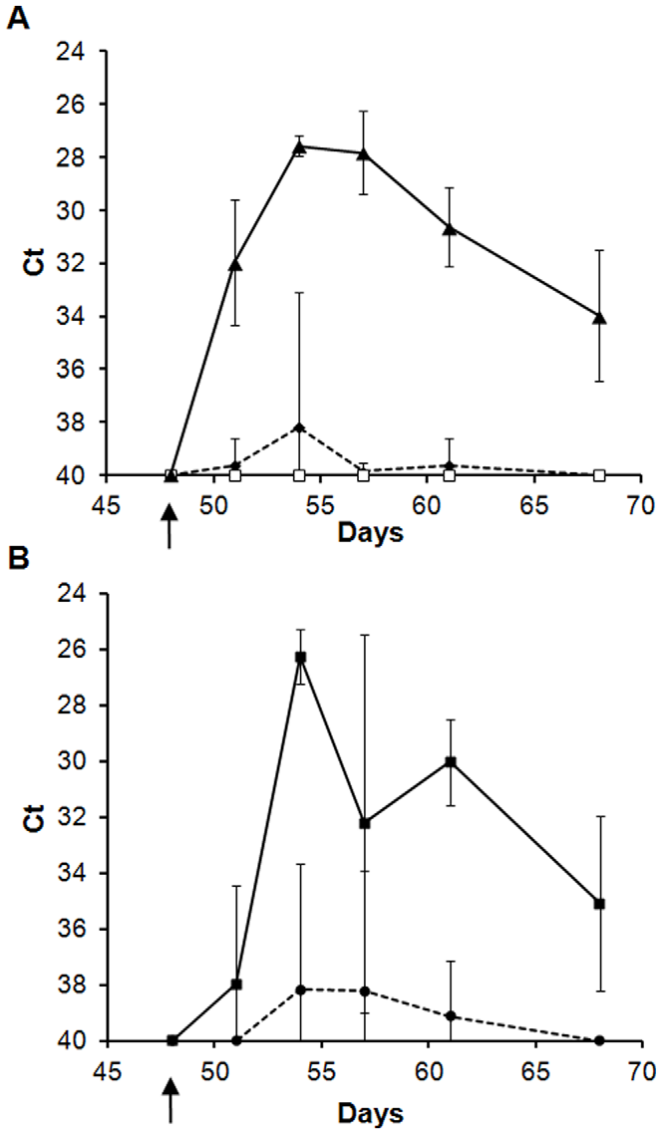
Detection of viraemia in vaccinated animals following challenge with virulent BTV. BTV genomic RNA was detected in blood samples by quantitative RT-PCR follow throughout the experiment. None of the animals had any detectable viral RNA prior to challenge. Each Ct values represented is the average of all animals in each group, no adjustments or omissions have been made. **A**. Animals were challenged with virulent BTV-1. Post- challenge, none of the sheep vaccinated with bivalent BTV-1 & BTV-4 VLPs vaccine (Group B) had detectable viraemia, one sheep vaccinated with monovalent BTV-1 VLPs (Group A) had detectable viraemia and all saline vaccinated control animals (Group D) had detectable viraemia. The different groups are indicated; Group D (▴), Group A (⧫) and Group B (□). **B.** Animals were challenged with virulent BTV-4. All control animals (Group E) developed viraemia, while 2 of the 6 animals vaccinated with bivalent BTV-1 & BTV-4 VLPs vaccine (Group C) had detectable viral dsRNA. The different groups are indicated; Group E (▪) and Group C (•).

Although only 1/20 VLP vaccinated, challenged animals (sheep C5) showed any detectable clinical signs we were interested to determine whether there was any detectable viral RNA in challenged animals. Whole blood samples from all animals before and after challenge were taken (as Materials and Methods) and presence of viral RNA was determined by quantitative RT-PCR. The background cycle threshold (Ct) with the RT-PCR test for all samples was 40. Animals vaccinated with saline and adjuvant and challenged with virulent BTV-1 had individual Ct values ranging from 34.62 to 26.55 from day 51 to day 68 with peak viraemia on day 54, where the average Ct was 27.57 (Fig. 5A). For the animals vaccinated with monovalent or bivalent BTV-1 VLPs and challenged with the same virus (Groups A and B) only 1 animal (sheep A2) had a Ct different to background. This was the same sheep that had failed to elicit neutralising or group specific antibodies and it had detectable viral RNA from day 51 to day 61. Although, viraemia and Ct values recorded for this animal mirrored the viraemia for the control animals challenged with same virus BTV-1 (Table S1), it had no clinical signs of bluetongue disease. All animals in vaccinated with the bivalent VLPs and challenged with virulent BTV-1 (Group B) had no detectable viraemia following virulent virus challenge (Fig. 5A).

All animals had a Ct value of 40 prior to challenge with virulent BTV-4 indicating that there was no detectable BTV dsRNA (Fig. 5B). In the control animals, detectable viral RNA peaked at day 54, down at day 57 and increased again at day 61 (Fig. 5B). The variation observed in control animals was due to the onset of viraemia occurring on different days for each animal; no omission or adjustment of the data points were made, to allow a fair comparison between treatment groups. Circulating BTV RNA was detected in sheep E3 and E1 on days 51 and 54, respectively. In sheep E2, detectable RNA was delayed by 10 days in comparison to the other control animals with viral RNA first detected on day 61 (Table S1). All control animals had Ct values below 40 on day 61 and day 68 (Fig. 5B). The range of Ct values through this viraemic period were from 27.67 (sheep E3, day 57) to 38.17 (sheep E2, day 68). Two of the six sheep vaccinated with BTV-1 and BTV-4 VLPs and challenged with virulent BTV-4 had detectable viral RNA (sheep C5 and C6). Sheep C5 had detectable RNA from day 51 until its death on day 65 with Ct values in the range from 29.4 to 33.97 (Table S1). Sheep C6 was positive on a single day (day 61) with a Ct value of 35.62 but samples before and after this from the same sheep were negative. All other sheep vaccinated with the bivalent VLPs did not develop viraemia after challenge with virulent BTV-4 and had Ct values below the limit of detection (Fig. 5B).

## Discussion

Previous studies have demonstrated the assembly of BTV-1 and BTV-4 VLPs [21], [22]. However, prior to this study there has been no investigation of the immunogenicity of these VLPs in sheep or whether VLPs for these two serotypes could be combined to make a bivalent vaccine. In this study we have addressed these questions.

Initially, antibodies to group specific antigen VP7 were used as an indication of immune response in vaccinated sheep. Two commercial ELISA kits were used for this purpose, one double recognition ELISA kit (Ingenasa) and one competitive ELISA kit (IDVET). The double recognition ELISA kit gave an earlier indication of immune response to VLPs, but the competitive ELISA was more indicative of overall immune response of the animal to the vaccine. The sensitivity of both of the ELISA kits to determine sero-conversion of animals to the group specific antigen (VP7) requires greater comparison due to the different results in regards to the time of sero-conversion from the same serum sample. This is most evident for sheep A2, which was positive from day 10 for the double recognition ELISA, but remained negative in the competitive ELISA until after challenge. This sheep was also the only animal vaccinated with BTV-1 VLPs that had detectable RNA for the challenge virus by RT-PCR, and the only animal with a background level of serum neutralisation assay to BTV-1 on day 30. Despite these findings sheep A2 was completely protected from clinical signs including pyrexia on challenge. This is a marked contrast to control animals challenged with the same virus (Group D). The detection of limited virus replication in a small proportion of vaccinated animals is consistent with current commercial inactivated vaccines to bluetongue virus serotype 8 [23], [24]. Interestingly, in these studies virus replication on challenge was also correlated with generally poor immune response to the vaccine [23] or lack of neutralising antibodies [24].

Challenge experiments with sheep vaccinated with bivalent VLP vaccine and challenged with BTV-4 resulted in one vaccinated animal (C5) developing prolonged viraemia and clinical signs consistent with BTV infection prior to death. One other animal (C6) was positive for BTV by RT-PCR on one day following challenge (day 61) but developed no clinical signs and no persistent viraemia. These results were surprising because a previous study with VLPs of a different European isolate (BTV-2) with similar amount of antigens in a different breed of sheep and challenge virus used as a monovalent vaccine had resulted in complete abrogation of clinical signs and detectable virus replication in all vaccinated animals following challenge [21]. Quality control by SDS-PAGE and electron microscopy with the BTV-1 and BTV-4 VLPs had not revealed a difference between serotypes in the quality of VLPs used for immunisation. With the sample sizes used in this study it is unclear whether the reason for the failure of the bivalent vaccine to protect animal C5 was due to interference in immune response to BTV-4 VLP caused by immunodominance of BTV-1 VLP, or whether this animal was just extremely poor at responding to the vaccine, as for sheep A2 and C6, or was particularly susceptible to infection with BTV. Previous studies have noted a marked difference in susceptibility between breeds, species and individuals for development of clinical disease to BTV [4], [23], [25]. It is interesting to note that none of the control animals challenged with the same virus died, suggesting that there was something unusual in the ability of this animal to respond to BTV infection. It could be that heightened clinical signs in these two BTV-4 challenged sheep were related to route of inoculation. There is some evidence that intradermal delivery of challenge virus results in clearer disease [26]. However, this does not explain the difference between the sheep that died (C5) and the controls challenged with the same virus. Other studies with monovalent BTV VLPs and intradermal challenge indicate that protection is achieved even with this route for the virulent challenge virus [27]. One further possibility is that the BTV-4 VLPs were ineffective as an immunogen and the protection from BTV-4 challenge was due to cross-protection from BTV-1 VLP immunisation. This is unlikely, as previous studies indicate that immunological protection from BTV infection with monovalent vaccines is usually serotype specific [20], [28]. There is some limited evidence of cross protection against phylogenetically related serotypes [20] but BTV-1 and BTV-4 do not have a close relationship in this respect. There is also one recent report suggesting cell-mediated protection from severe disease from a heterologous seroptype following use of an inactivated vaccine [29]. This is not generally consistent with other observations on immunity to bluetongue [28]. However, given the unexpected results in this study, future studies should include a specific control group for the monovalent BTV-4 VLP immunogen and larger groups of animals in order to assess the potential for cross-protection and immunodominance in BTV cocktail vaccines.

## Materials and Methods

### Viruses and cells

Bluetongue virus neutralisation tests were carried out using BSR cells (a derivative of baby hamster kidney cells, ATCC) cultured in Dulbecco's modified Eagle medium (DMEM) supplemented with 10% fetal calf serum. Cells were incubated in a humidified chamber at 37^o^C with 5% CO_2_. Challenge viruses were a virulent BTV-4 challenge strain, supplied by Merial (virus titre not supplied), and BTV-1 ALG2006/01 E1/BHK2 (3.8x10^7^ TCID_50_/ml) supplied by CISA-INIA. The development of the two different system to produce recombinant baculoviruses for the production of BTV-1 VLPs and BTV-4 VLPs have been previously described [21], [22]. Briefly, all four proteins in the BTV-1 VLP were expressed from 4 different loci throughout the baculovirus genome and were all derived from BTV-1 RSA. In comparison, the 4 proteins for the BTV-4 VLP are expressed from 2 different loci (*ph* and *p10*), the outer capsid proteins (VP2 and VP5) are derived from BTV-4 Corsica and the inner capsid proteins are from BTV-10 (VP7) and BTV-17 (VP3). The recombinant baculoviruses were cultured in *Sf*9 (*Spodoptera frugiperda*, ATCC) cells in SF900II serum free medium (Invitrogen) at 28°C.

### Preparation of BTV VLP vaccines

VLPs for immunisation were prepared as described [21], [22], with the exception that 5 mM EGTA was substituted for 10 mM EDTA in the lysis buffer and samples were not sonicated. The presence of all four BTV proteins (VP2, VP3, VP5, VP7) in purified VLPs was validated by SDS-PAGE and western immunoblot using specific antibodies, and the morphology of the particles was confirmed by electron microscopy as described previously [21], [22], [30]. Prior to immunisation VLP were stored at 4°C.

### Vaccination and Challenge experiments in sheep

All the procedures described in this study were carried out according to Spanish and European regulations on animal welfare and were approved by the animal experimental committee from the Complutense University of Madrid (Committee for Animal Experimentation, UCM, 2003), in line with the National law (Royal Decree 1201/2005 and Law 32/2007), which are in accordance with the Directive of the Council of the European Communities (86/609/EEC). For the vaccination and challenge study 26, 7–8 month old healthy male Merino sheep were used. Animals were in-housed in the VISAVET biosafety level 3 facilities in the Complutense University of Madrid, with *ad libitum* food and water. Prior to vaccination challenge study, animals were tested for BTV and BTV antibodies. Animals were acclimatised to the experimental facilities 7 days prior the first vaccination and their health status evaluated to provide a baseline for experiments. Five groups, of 3–8 animals were segregated randomly. Groups D and E (3 animals per group) were control animals and were inoculated with saline plus SEPPIC adjuvant (Montanide ISA 206 VG consisting of mannide oleate and mineral oil). Group A (8 animals) was vaccinated with a monovalent BTV-1 VLP vaccine and groups B and C (each of 6 animals) were vaccinated with the bivalent BTV-1 and BTV-4 VLP vaccine. The adjuvant was the same for all vaccines. Animals were vaccinated subcutaneously on day 0 in the axillary region, and a second booster vaccination was given on day 20 by the subcutaneous route on the back of the neck. On day 48, groups A, B and D were challenged with 1 ml of BTV1 ALG2006/01 E1/BHK2 (3.8×10^7^ TCID_50_/ml, provided by CISA-INIA) by administrated to the jugular vein. Groups C and E were challenged with 1 ml virulent BTV-4 virus (supplied by Merial) delivered via intradermal route as described [21]. At the end of the experiment, days 69 and 75, all animals were euthanased.

### Clinical observations and sample collections from infected sheep

Sheep were observed after challenge with virulent BTV for the development of classical bluetongue disease symptoms, all symptoms were recorded. The rectal temperature was measured (“Krusse® Instant digital thermometer for domestic animals), on days 47 until day 68 as an indictor of BTV infection. Blood samples were collected from sheep to monitor antibody response in plain tubes on days 0, 10, 20, 35, 42, 48, 53, 58, 68 and prior to euthanasia (day 74 and 75) and heat inactivated for 30 minutes at 56°C. Whole blood samples were also collected into ethylene diaminetetraacetic acid (EDTA) tubes on days 48, 51, 54, 57, 61, 68, and prior to euthanasia to enable the RT-PCR detection of the circulating virus.

### Group Specific ELISA assays

Antibody responses to BTV group-specific antigen VP7 were assessed using the ID Screen® Bluetongue Competition ELISA kit (ID VET, France) and the BTV DR 12.BTV.K0 double recognition ELISA (Ingenasa, Ingezim, Spain) according to the manufacturers protocols.

### RNA extraction and RT-PCR

Extraction of viral RNA from blood samples was carried out using the NucleoSpin® RNA II kit (Macherey-Nagel, Madrid) according to the manufacturer's instruction. Quantification of BTV RNA was carried out using primers specific for BTV segment 5 on a Mxpro3000® thermocycler (Stratagene) as described previously [31].

### Detection of neutralisation antibody response in the sera of vaccinated animals

The development of neutralising antibody response to the BTV VLPs was assessed in BSR cells by plaque reduction and serum neutralisation with 50 TCID_50_ as described [21], [32], [33]. All dilutions were preformed in triplicate and the assay repeated at least twice.

## Supporting Information

Table S1 **The Ct values determined by real time RT-PCR for animals challenged with either BTV-1 or BTV-4.** Animals with an A, B or C were challenged with virulent BTV-1. Animals with a D or E were challenged with virulent BTV-4. Day 48 is the day of challenge. A Ct of 40 was considered to be negative for BTV dsRNA.
(DOC)
