# Supplementary material for: Characterization of Protection Afforded by a Bivalent Virus-Like Particle Vaccine against Bluetongue Virus Serotypes 1 and 4 in Sheep
Source: PLoS One. 2011 Oct 21;6(10):e26666. doi: 10.1371/journal.pone.0026666 (PMC3202233; doi:10.1371/journal.pone.0026666)
Supplement: Table S1 — The Ct values determined by real time RT-PCR for animals challenged with either BTV-1 or BTV-4. Animals with an A, B or C were challenged with virulent BTV-1. Animals with a D or E were challenged with virulent BTV-4. Day 48 is the day of challenge. A Ct of 40 was considered to be negative for BTV dsRNA. (DOC) [file pone.0026666.s001.doc]

|  | Ct values | | | | | |
| --- | --- | --- | --- | --- | --- | --- |
|  | Days post challenge | | | | | |
| Animal | 48 | 51 | 54 | 57 | 61 | 68 |
| 1A | 40 | 40 | 40 | 40 | 40 | 40 |
| 2A | 40 | 37.08 | 25.53 | 38.99 | 37.12 | 40 |
| 3A | 40 | 40 | 40 | 40 | 40 | 40 |
| 4A | 40 | 40 | 40 | 40 | 40 | 40 |
| 5A | 40 | 40 | 40 | 40 | 40 | 40 |
| 6A | 40 | 40 | 40 | 40 | 40 | 40 |
| 7A | 40 | 40 | 40 | 40 | 40 | 40 |
| 8A | 40 | 40 | 40 | 40 | 40 | 40 |
| 1B | 40 | 40 | 40 | 40 | 40 | 40 |
| 3B | 40 | 40 | 40 | 40 | 40 | 40 |
| 3B | 40 | 40 | 40 | 40 | 40 | 40 |
| 4B | 40 | 40 | 40 | 40 | 40 | 40 |
| 5B | 40 | 40 | 40 | 40 | 40 | 40 |
| 6B | 40 | 40 | 40 | 40 | 40 | 40 |
| 1C | 40 | 40 | 40 | 40 | 40 | 40 |
| 2C | 40 | 40 | 40 | 40 | 40 | 40 |
| 3C | 40 | 40 | 40 | 40 | 40 | 40 |
| 4C | 40 | 40 | 40 | 40 | 40 | 40 |
| 5C | 40 | 40 | 30.27 | 29.4 | 31.27 | 33.97 * |
| 6C | 40 | 40 | 40 | 40 | 35.62 | 40 |
| 1D | 40 | 30 | 28.92 | 26.55 | 29.87 | 31.84 |
| 2D | 40 | 31.32 | 31.88 | 29.58 | 32.36 | 36.7 |
| 3D | 40 | 34.62 | 30.29 | 27.34 | 29.68 | 33.45 |
| 1E | 40 | 40 | 34.00 | 29.02 | 28.4 | 31.89 |
| 2E | 40 | 40 | 40 | 40 | 31.49 | 38.17 |
| 3E | 40 | 33.92 | 29.45 | 27.67 | 30.22 | 35.23 |

* Ct for animal 5C is from day 65 not 68. because the animal died on day 66
